# Effects of prophylactic antibiotic-treatment on post-surgical recovery following intraperitoneal bio-logger implantation in rainbow trout

**DOI:** 10.1038/s41598-020-62558-y

**Published:** 2020-03-27

**Authors:** Per Hjelmstedt, Henrik Sundh, Jeroen Brijs, Andreas Ekström, Kristina Snuttan Sundell, Charlotte Berg, Erik Sandblom, Jennifer Bowman, Daniel Morgenroth, Albin Gräns

**Affiliations:** 1 Department of Animal Environment and Health, Swedish University of Agricultural Sciences, Skara, SE-532 31 Sweden; 2 Department of Biological and Environmental Sciences, University of Gothenburg, Gothenburg, SE-405-30 Sweden; 3 Swedish Mariculture Research Center, Centre for Sea and Society at University of Gothenburg, Gothenburg, SE-405-30 Sweden

**Keywords:** Immunology, Physiology

## Abstract

Bio-logging devices can provide unique insights on the life of freely moving animals. However, implanting these devices often requires invasive surgery that causes stress and physiological side-effects. While certain medications in connection to surgeries have therapeutic capacity, others may have aversive effects. Here, we hypothesized that the commonly prescribed prophylactic treatment with enrofloxacin would increase the physiological recovery rate and reduce the presence of systemic inflammation following the intraperitoneal implantation of a heart rate bio-logger in rainbow trout (*Oncorhynchus mykiss*). To assess post-surgical recovery, heart rate was recorded for 21 days in trout with or without enrofloxacin treatment. Contrary to our hypothesis, treated trout exhibited a prolonged recovery time and elevated resting heart rates during the first week of post-surgical recovery compared to untreated trout. In addition, an upregulated mRNA expression of TNFα in treated trout indicate a possible inflammatory response 21 days post-surgery. Interestingly, the experience level of the surgeon was observed to have a long-lasting impact on heart rate. In conclusion, our study showed no favorable effects of enrofloxacin treatment. Our findings highlight the importance of adequate post-surgical recovery times and surgical training with regards to improving the welfare of experimental animals and reliability of research outcomes.

## Introduction

Across a wide range of disciplines, approximately 11.5 million experimental animals were used for research purposes within the member states of the European Union in 2011^1^. While the vast majority of these animals are rodents (~80%), the proportion of ectotherms, including teleost fishes, is rapidly increasing^1^. One expanding area within experimental fish research concerns the use of novel implantable electronic tags (*e.g*. bio-logging and bio-telemetric devices). The recent technological developments and use of bio-logging and bio-telemetric devices in aquatic organisms has been proposed to open up a ‘panoramic window into the underwater world’^2^. The use of these devices in freely swimming fish allow the continuous collection of high-resolution physiological and behavioural data (*e.g*. heart rate, blood flow and muscle activity) over long periods of time^3–9^. Moreover, data from implanted fish swimming amongst conspecifics (*i.e*. focal animals) can provide important insights into relationships between physiological and behavioral traits across different social contexts in both natural and aquaculture settings^3,10^. However, there are still challenges associated with the use of these implants in order to produce reliable high-quality data, as well as to safeguard the health and welfare of the experimental animal in accordance with the 3 R guidelines.

First of all, introducing a foreign body into an animal may lead to expulsion or encapsulation of the implant, and secondly, the protective barrier of the epithelium is breached during surgery where after the wound can act as an entry-point for pathogens, which will increase the risk of infection and immune reactions^11–14^. The wound repair process starts immediately, as the infliction of a wound initiates local inflammation and tissue repair mechanisms^15^. When a bacterial infection occurs, a systemic inflammatory response is induced. The pathogen is detected by the innate immune system, which elicits a cascade of adaptive immune responses in a complex network involving pro- and anti-inflammatory cytokines to neutralize the threat^16^. When a fish experiences such a systemic immune response it is generally linked to an activation of the hypothalamic-pituitary-interrenal axis (HPI-axis) and the release of corticosteroid stress hormones such as cortisol^17,18^. However, a fish undergoing a surgical procedure will additionally experience a combination of other stressors (*e.g*. noxious stimuli, handling, air exposure, anesthetics), which may also have aversive effects on the welfare of the fish and the quality of the obtained data.

In human and veterinary medicine, a wide range of drugs such as antibiotics, analgesia, general- or local anesthesia are commonly used for pain and wound healing management, as well as to facilitate a speedy recovery and minimize the aversive effects of surgery. In experimental fish research, the use of such drugs is still comparably low^19^. However, as the public concern regarding fish welfare is increasing and technical developments have made implantable electronic tags widely available, the use of post-surgical drug treatments is also increasing in fish research^20–22^. When using implantable devices, the fish is normally released back into the wild or into a large school of fish in an aquaculture setting for a relatively long period of time. In such studies, the assessments of wound healing, inflammatory responses or general health of the individual fish is difficult. Thus, treatment with antibiotics is sometimes used to reduce the risk of post-surgical infections when individual monitoring is impossible^20,23^. However, despite the common use of antibiotic treatment in mammals^24^, little is known about the therapeutic efficiency, preferential administration routes and dosages for fish. Following the recommendation of veterinarians, the antibiotic enrofloxacin has frequently been used to prevent post-surgical infections in fish^3,7,25–27^. It is a broad-spectrum fluoroquinolone antibiotic that has been shown to efficiently reduce mortality (10-fold) in farmed adult and juvenile Atlantic salmon (*Salmo salar*) diagnosed with the bacterial disease furunculosis^28,110^. Despite the usefulness of prophylactic treatment with enrofloxacin to survive a bacterial infection, the effectiveness in fish subjected to experimental surgical protocols remains unknown.

Obviously, minimizing the risk of infections and allowing the fish an adequate recovery period to recover from the stress following surgery is important to safeguard fish welfare and to obtain unbiased data that are representative of the population. However, it can be difficult to determine when a fish is unstressed and no longer affected by the surgery. Traditionally, measurements of circulating levels of plasma cortisol from whole body- or blood sampling have been used as a proxy for measuring stress in fish, but this method requires the invasive collection of blood or tissue^29^. Novel alternative techniques are available and have been shown to provide robust data on cortisol levels, ranging from non-invasive sampling of faeces, urine or water-borne cortisol to more invasive sampling of fin tissue, mucous or scales^30,31^. However, all of these techniques require the collection of blood, water or tissue and will consequently only provide a “snapshot” of the recovery period or level of stress. As an alternative to measurements of cortisol, recent studies show a strong, significant relationship between cortisol and heart rate of fish responding to various acute stressors in aquaculture^3^, as well as during recovery from stress^4^. Therefore, by analysing heart rate during recovery, we can determine when fish have fully recovered from post-surgical stress as a low, stable resting heart rate coincides with the low levels of circulating plasma cortisol commonly associated with an ‘unstressed’ fish^3,4,29^.

The aim of this study was to investigate the effects of pre-surgical prophylactic treatment with enrofloxacin on post-surgical recovery in freely swimming adult rainbow trout implanted with heart rate bio-loggers. Specifically, we hypothesized that enrofloxacin-treatment would decrease the prevalence of infection and reduce the post-surgical recovery period. To address these hypotheses, we quantified local (visually assessed) and systemic inflammation markers (*i.e*. expression of key cytokines in the head kidney), as well as a range of primary and secondary stress indicators (*i.e*. heart rate, plasma cortisol and hematological variables) in rainbow trout with and without prophylactic enrofloxacin-treatment.

## Materials and Methods

### Animals

Rainbow trout (*Oncorhynchys mykiss*, Walbaum 1792) of mixed sexes were obtained from Vänneåns fiskodling (Knäred, Sweden) and transported to the Department of Biological and Environmental Sciences, University of Gothenburg. The fish were held in a 2000L tank supplied with recirculated, aerated freshwater maintained at 10 °C with a 12:12 hour photoperiod at a density of 15 kg m^−3^. Fish were allowed to acclimatize for at least three weeks before the experiments. The experimental procedures were approved by the ethical committee on animal research in Gothenburg, Sweden (Gothenburg animal testing ethics committee, ethical permit 2013-177) and all experiments were performed in accordance with relevant guidelines and regulations.

### Surgical procedure

Fish were individually anaesthetized in 10 °C freshwater containing MS-222 (150 mg l^−1^, ethyl 3-aminobenzoate methanesulphonate) buffered with 300 mg l^−1^ NaHCO_3_ in a 25 L bucket. When opercular movements ceased, the fish were transferred to a surgery table where they were placed on a water-soaked foam. Anesthesia was maintained during surgery by flushing aerated water containing MS-222 (100 mg l^−1^) and NaHCO_3_ (200 mg l^−1^) over the gills. Unless otherwise stated, all chemicals were purchased from Sigma-Aldrich Inc., St Louis, Missouri, USA.

Sterile Gammex PF (Ansell, Malmö, Sweden) gloves were used throughout surgeries and the instruments were thoroughly cleaned and rinsed in 70% ethanol and left to dry in air between surgeries. Iodine (Jodopax vet. Pharmaxim Sweden AB, Helsingborg, Sweden) diluted to 4 ml l^−1^ was applied to the skin of the fish before a ~4 cm mid-ventral incision was made between the pectoral and pelvic fins. A pit-tag (Passive Integrated Transponder, 12 mm, Oregon RFID, Portland, Oregon, USA) was first inserted into the abdominal cavity to allow individual identification. Bio-loggers (DST milli-HRT, Star-ODDI, Gardabaer, Iceland) were then placed into the abdominal cavity of 36 fish and anchored to their abdominal muscle with a 3-0 sterile monofilament non-absorbable Prolene suture (Ethicon, LLC, Puerto Rico, USA). The bio-logger enabled measurements of heart rate and body temperature, and was positioned in proximity of the pericardium to optimize signal strength and quality as described previously^3^. As the levels of investigated blood-borne variables can be highly variable, six additional fish were implanted with identical dummy loggers to increase statistical power in the analysis of these variables. The edges of the wound were powdered with antibacterial (Bacibact, Orion pharma, Espoo, Finland) and antifungal powder (Pevaryl 1%, McNeil Sweden AB), where after the wound was closed with 3-4 interrupted sutures (Prolene 3-0 sterile monofilament) and covered with Orabase paste (ConvaTec Inc, Deeside, UK). The surgical procedure took approximately 15 minutes and was performed simultaneously by two surgeons with different levels of experience.

### Experiment protocol and bio-logger configuration

Prior to surgery, half of the fish were randomly selected and given an intramuscular injection of enrofloxacin (10 mg kg^−1^ bodyweight, Baytril Vet. 25 mg ml^−1^, Bayer Animal Health GmbH, Leverkusen, Germany) above the lateral line posterior to the anal fin^3^. This particular route of administration was selected as it allows for the quick administration of a tightly controlled dose of enrofloxacin without the need for additional implants (*e.g*. slow-releasing implants). This group is hereafter referred to as the *ab-treated* group (mass: 710 ± 75 g, n = 21). The other half of the fish were handled identically but did not receive any antibiotic treatment (*untreated* group; mass: 696 ± 78 g, n = 21). After surgery, all fish were placed in a tank similar to the holding tank (*e.g*. 2000L, fish density 15 kg m^−3^) supplied with recirculating, aerated freshwater maintained at 10 °C with a 12:12 h photoperiod and left for 21 days. During the course of the experiment, fish were fed twice a week with commercial trout pellets (size 4, Protec Trout pellets, Skretting, Stavanger, Norway). Feeding was kept to a minimum to avoid a reduction of water quality due to leftover pellets, and faeces and unconsumed feed was flushed out of the aquaria once per week. To monitor the status of the wound and overall health of instrumented fish during the recovery period, a submersible camera (Sony Exmor R Steadyshot) was used to inspect the fish during the feeding events.

The bio-loggers sampled heart rate for 6 sec with a frequency of 100 Hz (*i.e*. 600 measurements) every 10 min. In addition, at 4, 11 and 17 days post-surgery, a 6 sec ECG recording was sampled (at midnight) to allow for subsequent evaluation of the signal quality and robustness of the heart rate recordings.

### Sampling procedures, cortisol and mRNA analyses

After 21 days, the fish were quickly dip netted and anaesthetized in water containing 12 mg l^−1^ metomidate hydrochloride (Aquacalm, Western Chemical Inc, Ferndale, US). A blood sample of 1 ml was immediately drawn from the caudal vessels using a 1 ml heparinized syringe. Fish were then euthanized with a blow to the head, weighed, and the surgical wound was photographed for subsequent analysis of the wound healing (see below for details). The blood was analysed for haematocrit as the fractional red cell volume (%) following centrifugation in duplicate 80 µL microcapillary tubes at 10 000 rpm for 5 min. The remaining blood sample was immediately centrifuged in 1.5 ml Eppendorf tubes (5 min, 10 000 rpm) and the aliquot plasma was transferred to 1 ml tubes and stored at −80 °C for later cortisol analysis.

Blood plasma cortisol levels were determined using a radioimmunoassay described by Young^32^ using cortisol antibody (dilution 1:3000, Code: S020; Lot: 1014-180182, Guildhay Ltd, Guildford, Surrey, UK) validated by Sundh^33^. 3H-cortisol hydrocortisone-[1,2,6,7-3H(N)] were used as tracer (NET 396, NEN Life Sciences Products, USA) and hydrocortisone (Sigma-Aldrich, St. Louis, USA) was used as cortisol standards. The radioactivity was determined with a β-counter, Wallac 1409 liquid scintillation counter (Wallac, Turku, Finland).

The head kidney was dissected out and placed in RNAlater (Ambion, Austin, Texas), kept at 4 °C for 24 h and then stored at −80 °C until analysis. RNA from 15–20 μg of each sample was homogenized and extracted using RNeasy Plus Mini (Qiagen GmbH, Hilden, Germany) following the manufacturers protocol. Due to high amounts of DNA in the tissue, RNase-Free DNase Set (Qiagen) was used to avoid reduction of RNA yield and quality. RNA concentrations were quantified using a NanoDrop 2000c spectrophotometer (Thermo Scientific, Waltham, Massachusetts) and diluted to 1000 ng μl^−1^, where after cDNA was synthesized using iScript cDNA Synthesis Kit (Bio-Rad, Hercules, California) in a Bio-Rad MyCycler (RNA template concentration 2.5 ng μl^−1^). mRNA transcript levels of the pro-inflammatory cytokine tumour necrosis factor alfa (TNFα) and anti-inflammatory cytokine transforming growth factor beta (TGFβ) was obtained using qPCR with SsoAdvanced Universal SYBR Green Supermix (Bio-Rad) (5 μl) using 0.5 μl primers (0.5 μM) and run in duplicates at 61 °C with a total amount of 2,5 ng cDNA (4 μl template, 10 μl final reaction volume) (Bio-Rad CFX Connect Real-Time System, Bio-Rad CFX manager 3.1) including NTC and control samples excluding iScript Reverse Transcriptase. The efficiency of the rainbow trout specific primers was determined using dilution series (t/2, 50 – 1.5625 ng) (Supplementary Table S1). Elongation factor 1 alfa (ELF1α) was used as reference gene (primer concentration 0.3 μM) and gene expression was determined using the ΔC_T_-method. Furthermore, to enable the determination of how the relative expression of the cytokines related to that of completely uninstrumented fish, 10 fish housed in a separate similar tank were sacrificed, sampled in an identical manner as the two experimental groups and used as negative reference group.

### Analytical method and calculations

The bio-logger heart rate data was retrieved using the associated Communication Box (Star-ODDI, Gardabaer, Iceland). The software Mercury v 4.28 was used to extract the heart rate data and to generate measurement points, where only the highest graded (*i.e*. grade 0) heart rate recordings on a four-grade scale (*i.e*. grades 0–4) were used in this study, which represented 63 ± 2% of recorded data. This ensured that subsequent analyses were based on highly accurate measurements, as the measurement error associated with grade 0 recordings has been demonstrated to be <1 beat per minute (bpm)^4^. The heart rate recordings were analyzed for the daily mean heart rate (*i.e*. includes periods of spontaneous activity and tachycardia following feeding) and resting heart rate. Resting heart rate was defined as the 20^th^ percentile of the daily heart rate for each individual, which is the method suggested for determination of standard metabolic rate in fish^34^ and a slight modification of the method used by Brijs *et al*.^3,4^.

The site of incision and suture points were evaluated according to Wagner^35^ where redness of the suture entry and exit points of the first and last stitches, as well as the anchoring points (a total of six points), were blindly and independently rated by two evaluators using a binary scale 1 (inflammation) or 0 (no inflammation). In addition, the level of inflammation at the incision was evaluated on a 6 point scoring scale. Both scores were then summarized to obtain a final inflammation index score (maximum score: 12) as described by Wagner *et al*.^35^.

### Statistics

Statistical analyses were performed using SPSS version 24.0 (IBM Corp. Released 2016. IBM SPSS Statistics for Windows, Version 24.0. Armonk, NY: IBM Corp) and all data are reported as means ± SEM. To describe the temporal changes in heart rate during the 3-week postsurgical recovery period, a linear mixed model with Toeplitz repeated covariance matrix (*i.e*. the lowest Akaike’s Information Criterion) was used. The model was run separately on the three weeks post-surgery to avoid missing potential transient effects of the antibiotic treatment. For all models, individuals were set as subject variables and days post-surgery as the repeated variable. Each analysis was performed separately using the daily mean heart rate or the resting heart rate as the dependent variables. In the models, recovery time (day 1-21), experimental group (ab-treated and untreated), surgeon (1 and 2) and their interactions were included as fixed effects. If no interacting effects were observed, they were excluded from the models.

To further explore the general effects of treatment on recovery time, the daily mean and resting heart rates was compared to the heart rate of day 21. Day 21 was selected prior to experimentation as we assumed that at this time point all fish should have fully recovered and stabilized following the surgical implantation of the heart rate bio-loggers. For the non-repeated variables (*i.e*. cortisol, haematocrit, inflammation indices and the expression of TGFβ), independent samples t-tests were used to identify statistical differences. We also performed a paired samples t-test to explore metabolic state through weight differences before and after the trial for each group, followed by a one-way ANOVA to detect potential differences between groups. To meet the assumption of normal distribution, cortisol values were transformed using the natural logarithm (ln). As the data for the expression of TNFα did not meet the assumption of normal distribution, a non-parametric Kruskal-Wallis H-test was used to statistically analyze this variable. Statistical significance was accepted at *P* ≤ 0.05.

## Results

Visual observations of fish before and after feeding events revealed no obvious differences between the swimming and feeding behaviour of fish in the ab-treated and untreated groups. Furthermore, we did not observe the presence of any impaired or unusual behaviors such as elevated levels of aggression, which is further supported by the lack of wounds openings, fin/body damage or mortality during the course of the experiment. Statistically significant reductions in body mass of 16 ± 3.6 g (~1.4%, t_20_ = 4.50, *P* < 0.001) and 10.2 ± 5.8 g (~2.3%, t_20_ = 1.78, *P* = 0.045) were observed at the end of the experiment in untreated and ab-treated fish, respectively. However, the observed reductions in body mass did not statistically differ between the two groups (F_1,40_ = 0.73, *P* = 0.396).

### Effects of surgery on heart rate, cortisol and hematocrit

**Figure 1:**
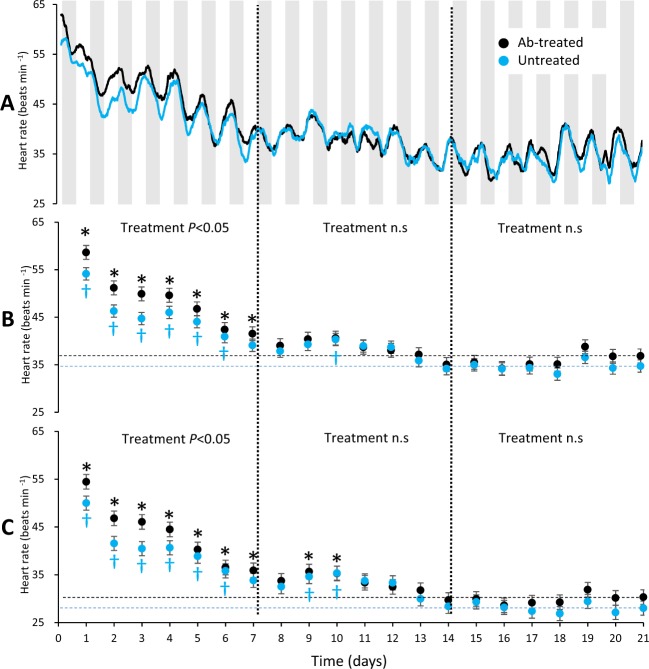
(**A**) Heart rate recordings for 21 days following the surgical implantation of heart rate bio-loggers (grey bars = lights off) in fish treated with antibiotics (ab-treated, black line) and untreated (blue line). (**B**) Post-surgical recovery assessed using daily mean heart rates and (**C**) resting heart rate (20^th^ percentile of daily mean). The black asterisk (*) and blue dagger (†) represents statistically significant (P < 0.05) elevations in heart rate compared to values on day 21 for ab-treated and untreated fish, respectively. Dashed black and blue lines highlight the heart rates of day 21 in ab-treated and untreated fish, respectively.

The mean heart rate of both groups were initially ~60 beats per minute (bpm), which steadily decreased during the first week and plateaued around 30–40 bpm (Fig. 1A). A significant treatment effect on heart rate was found, however, with heart rate being on average 3.7 (resting) and 3.8 (mean) bpm higher in the ab-treated fish during the first week (resting; F_1,27.06_ = 4.69, *P* = 0.039 and mean; F_1,27.86_ = 7.50, *P* = 0.011 respectively; Fig. 1B,C). After approximately three days, a clear diurnal pattern emerged with a ~10 bpm difference between day and night for both ab-treated and untreated fish (Fig. 1A). Overall, the post-surgical recovery of heart rate was faster in untreated fish, as heart rate was significantly elevated relative to day 21 for six days in the untreated fish and seven days in ab-treated fish (F_20,87.74_ = 35.46, *P* < 0.001, Fig. 1B, F_20,86.03_ = 29.66, *P* < 0.001, Fig. 1C). Although, with respect to heart rate, it was deemed that both untreated and treated fish had fully recovered from surgery on day 21, both groups exhibited a transient increase in heart rate on days 9-10 when compared today 21 (Fig. 1C).

**Figure 2:**
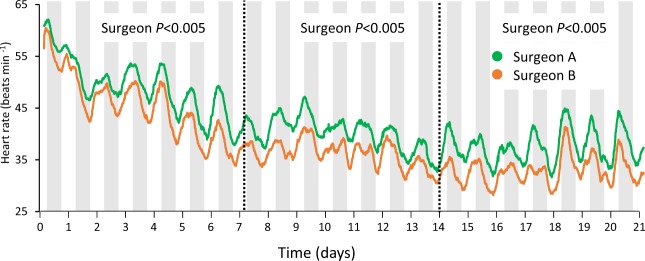
Recordings of heart rate showed a strong correlation to experience level of the surgeon. Throughout the entire three week trial period, the fish instrumented by the less experienced surgeon (surgeon A, n = 14, green line) had an elevated heart rate of 5.1 (resting) and 4.6 (daily mean) beats min^−1^ compared to the more experienced surgeon (surgeon B, n = 22, orange line).

**Figure 3:**
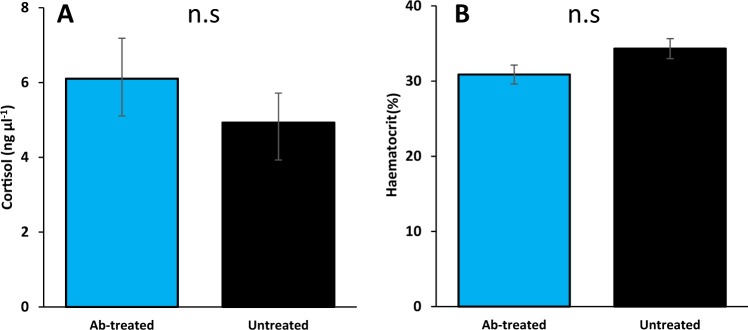
Haematocrit and blood plasma cortisol levels 21 days post-surgery. (**A**) Treatment with enrofloxacin had no effect on circulating blood plasma cortisol where levels were 6.1 ± 1.08 and 4.93 ± 0.79 ng μl^−1^ for ab-treated and untreated respectively 21 days post-surgery. (**B**) Haematocrit count was 30.9 ± 1.26% for ab-treated fish with no significant difference to the untreated group (34.3 ± 1.33%).

There was also a significant effect on heart rate depending on who performed the surgeries, whereby fish instrumented by the more inexperienced surgeon had heart rates 5.1 bpm (resting; F_1,32.50_ = 12.28, *P* < 0.005) and 4.6 bpm (mean; F_1,32.73_ = 11.66, *P* < 0.005) higher than fish instrumented by the more experienced surgeon (Fig. 2). This was not a transient effect, but lasted throughout the entire 21 day trial period and was present in both treatment groups. No interaction effect was found between treatment and surgeon in any of the analyses (*P* > 0.9). At the end of the 21 day recording period, both groups had plasma cortisol levels <10 ng ml^−1^ (Fig. 3A), and there were no significant differences between the groups (t_40_ = −0.839, *P* = 0.407). Haematocrit levels were also similar between groups (t_39_ = 1.87, *P* = 0.069, Fig. 3B).

### Inflammatory response

**Figure 4:**
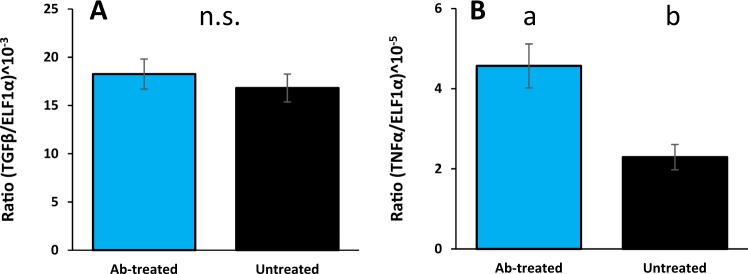
mRNA expression of key cytokines relative to housekeeping gene ELF1α from head kidney, sampled 21 days post-surgery. (**A**) No differences were found between groups for expression of TGFβ, n = 7, however, (**B**) the ab-treated group had a significantly increased expression of TNFα compared to the untreated group n = 6. (45.7 ± 5.48 versus 22.3 ± 3.17, (all values *10^−6^) respectively.

At the end of the trial, all fish were in seemingly good health, and no obvious fungal infections or signs of aggression (*i.e*. bite marks or fin damage) were found. Additionally, there were no signs of encapsulation or expulsion of the bio-loggers. The mean inflammation rating scored low in both groups and were not significantly different (1.71 ± 0.39 and 1.62 ± 0.38 for untreated and ab-treated fish, respectively, t_40_ = 0.177, *P* = 0.86). Similarly, the mRNA expression of TGFβ did not differ between the groups (t_12_ = −0.177, *P* = 0.863, Fig. 4B), but there was a significant, nearly doubled, upregulation of TNFα mRNA expression in ab-treated trout (χ2_1,12_ = 5.545, *P* < 0.019, Fig. 4A).

## Discussion

Contrary to our hypothesis, prophylactic ab-treatment following the surgical implantation of heart rate bio-loggers did not decrease the prevalence of infection or reduce the post-surgical recovery time. Instead, the ab-treatment induced potentially aversive effects on both the gradual recovery of heart rate following surgery, as well as the mRNA expression of the pro-inflammatory cytokine TNFα three weeks post-surgery. Since fish are ectotherms, both bioavailability and half-life of enrofloxacin are affected by water temperature^36^. The half-life of an intramuscular injection of enrofloxacin (10 mg kg^−1^) was ~85 hours in juvenile Atlantic salmon at 10 °C, however, the tissue depletion time of this compound has been shown to be species-specific and even longer in rainbow trout than in salmon^37,38^. Thus, the relatively long depletion time of this substance might explain why ab-treated fish displayed a significantly higher mean heart rate (*i.e*. 3.7–3.8 bpm) throughout the first six days post-surgery, as well as a more prolonged overall recovery time as both mean and resting heart rates required an extra day to return to baseline levels when compared to untreated fish. Importantly, as the difference in heart rate between untreated and ab-treated fish was similar for both resting and daily mean heart rate, this implies that behavioural differences (*e.g*. swimming activity) between groups does not explain this effect. Interestingly, following the recovery from the permanent implantation of the bio-logger, both groups of fish exhibited a transient increase in heart rate on days 9–10. Although the underlying reason for this response remains unknown, the presence of this response in both groups demonstrates that fish were able to behaviorally and/or physiologically respond in a similar manner to the unknown stimuli. This unexplained transient elevation in heart rate has also been documented in a previous study on single-housed rainbow trout exposed to buprenorphine in the same aquaria facilities^39^, and thus it may be beneficial for future studies to employ video and sound recording in experimental rooms to explain these seemingly random events.

An upregulation of the mRNA expression of TNFα in the head kidney was observed in ab-treated fish three weeks after surgery. Being a pro-inflammatory cytokine, TNFα is known to be involved in the acute phase reaction during infection, inflammation and/or vaccination^39,40^. This may indicate an inflammatory response. However, it should be kept in mind that mRNA will undergo post- transcriptional regulation to reach the functional protein. In rainbow trout, increased TNFα mRNA expression has been observed in absence of TNFα protein secretion^41^. Thus, the significance of increased TNFα mRNA levels should be interpreted with care. Furthermore, we found no evidence for a stress response of fish from either group three weeks after surgery, as levels of circulating plasma cortisol were within the expected range of unstressed trout (<10 ng μl^−1^ ^29^). In addition, hematocrit levels were normal (30–35%^42,43^) and there was a low prevalence of visual signs of inflammation around the wound^44^. Overall, these results suggest that the welfare of the fish was not impaired.

During the period following surgery, mean and resting heart rates were both elevated for at least 6 days. Previous field studies in aquaculture settings have shown that it takes at least three days for heart rate to fully recover from the stress associated with implantation of the same type of bio-loggers^3,26^. This discrepancy between field and laboratory studies can partly be explained by higher ambient temperature in the field studies. Indeed, wound healing rate can be different, where the warm water zebrafish (*Danio rerio*) have been shown to heal at a much faster rate than cold water species such as rainbow trout^45,46^. The water temperature was roughly 5 °C colder in our study compared to the other field studies, which could explain why those fish recovered faster. In the abovementioned aquaculture field studies both the daily mean and resting heart rate plateaued at higher levels in the field (∼55–60 bpm, daily mean). This too could be an effect of the higher temperature but it might also be that fish in aquaculture environments are exposed to a higher level of general stress or increased activity levels compared to laboratory housed animals. At 15 °C, heart rate of rainbow trout was lower in laboratory environment (32 bpm)^47^ compared to heart rates reported in field studies^3,26^, suggesting a situation where the laboratory environment allow for “real” resting levels, *i.e*. possibly lower than what would be seen in the field. Similar to previous studies, a clear circadian rhythm in heart rate was absent during the first ~3 days following surgery, which has been suggested to be an indicator for post-surgical stress and potentially reflects behavioural disturbances in swimming activity^3,48,49^.

Although the intraperitoneal implantation of a bio-logger is a relatively simple surgical procedure, the fish in our experiments are still subjected to a series of stressors, which include (i) capture of the fish by netting, (ii) exposure to an anesthetic agent^50^, (iii) 15 minutes of surgery, (iv) the presence of a foreign body within the abdomen, and (v) the reintroduction with conspecifics in a new environment during the recovery period^4,51,52^. Previous studies have demonstrated that these stressors may contribute towards the relatively long period of elevated heart rate observed in the present study^3,4,39^. For example, under similar laboratory conditions (*e.g*. fish held in recirculating aerated freshwater maintained at 10 °C with a 12:12 hour photoperiod at a density of ~15 kg m^−3^), heart rate of rainbow trout increased rapidly by ~25 beats min^−1^ following netting and took ~2 h to recover when alone^4^. In the same study, when trout were netted but instead grouped together at a density of ~15 kg m^−3^ during recovery, heart rate did not recover to pre-stress levels within 7 h^4^. Furthermore, in a separate study under the same conditions, when rainbow trout were anaesthetised using MS-222 and subjected to a similarly sized abdominal incision without the implantation of a bio-logger, heart rate of trout remained elevated by ~10 beats min^−1^ for 24 h after surgery^39^. In addition to the isolated effects of each stressor, repeated stress induced by multiple stressors have also been demonstrated to have a cumulative and long-lasting effect on heart rate of rainbow trout^3^. Thus, further refinement to the techniques associated with the implantation of bio-logging or bio-telemetric devices is warranted, as a reduction in post-surgical recovery time would be beneficial for both the wellbeing of the experimental animal and the outcome of the experiments. This is because stress compromises the ability for the fish to maintain homeostasis and potentially increases their vulnerability to infections which is somewhat problematic^53^. In addition, if a fish is exposed to a new stressor while recovering from an earlier stressor, their physiological or behavioural responses may not be representative of that of a healthy fish, which will consequently bias the results of the study^4,26^.

Interestingly, our results also clearly show that surgical training and experience play an important role in improving post-surgical wound healing and the welfare of experimental animals, as less experienced surgeons often need more time to perform the surgery and may close the wound too tight^54^. Consistently, surgical times were noted to be approximately a few minutes longer for the inexperienced surgeon and it also resulted in somewhat longer suture ends. Indeed, the difference in surgical experience accounted for an elevation in heart rate of ~5 beats min^−1^, which strongly suggests that refining surgical protocol may be more important than casual prophylactic use of antibiotics to facilitate fast recovery from instrumentation in fish^55^. However, prophylactic use of enrofloxacin may still be necessary in experiments conducted in environments where the risk of infection is significant and the possibility to monitor of fish welfare continuously is limited, and warrants further investigation. Our findings also highlight the importance that fish are given adequate post-surgical recovery times before the start of the experiments to avoid treatment bias.

## Conclusion

The present study highlights the importance of researchers being aware of the potential side-effects when exposing animals to a drug as part of the experimental protocol. The purpose of medication should be to provide the best possible care for the animal by minimizing health problems or other welfare issues, which in turn should lead to more reliable data in accordance with the 3R concept. However, as this study demonstrates, the side effects of the medication need to be examined as they can potentially impinge on the health and welfare of the experimental animal, as well as the reliability of the data. Our findings are not only important from a 3R perspective but also from an antibiotic resistance viewpoint, as unnecessary use of antibiotics should be avoided. Thus, future improvements in surgical protocols and training may be more beneficial for the experimental animals and research outcomes, especially considering the growing number of both individuals and species of fish used in experiments.

## Supplementary information

Supplementary information.

## Data Availability

The datasets generated and analysed during the current study are available from the corresponding author on reasonable request.
