# Supplementary material for: Effects of prophylactic antibiotic-treatment on post-surgical recovery following intraperitoneal bio-logger implantation in rainbow trout
Source: Sci Rep. 2020 Mar 27;10:5583. doi: 10.1038/s41598-020-62558-y (PMC7101407; doi:10.1038/s41598-020-62558-y)
Supplement: Supplementary file 1 — Supplementary information. [file 41598_2020_62558_MOESM1_ESM.pdf]

Effects of prophylactic antibiotic-treatment on post-surgical recovery following intraperitoneal bio-logger implantation in rainbow trout

Per Hjelmstedt[1]*, Henrik Sundh[2,3], Jeroen Brijs[1], Andreas Ekström[2], Kristina Snuttan Sundell[2,3], Charlotte Berg[1], Erik Sandblom[2], Jennifer Bowman[1], Daniel Morgenroth[2], Albin Gräns[1]

[1] Department of Animal Environment and Health, Swedish University of Agricultural Sciences, Skara, SE-532 31, Sweden

[2] Department of Biological and Environmental Sciences, University of Gothenburg, Gothenburg, SE-405-30, Sweden

[3] Swedish Mariculture Research Center, Centre for Sea and Society at University of Gothenburg, SE-405-30, Sweden

*per.hjelmstedt@slu.se

Protein	Primer name	Primer sequence (5'-3')	Amplicon size (bp)	Accession number	Efficiancy (%)
**TNFα**	TNFα FwOm	CAAGAGTTTGAACCTCATTCAG	208	AJ277604	102.7
	TNFα ReOm	GCTGCTGCCGCACATAAAG			
**TGFβ**	TGFβ FwOm	AGATAAATCGGAGAGTTGCTGTG	275	X99303	104.7
	TGFβ ReOm	CCTGCTCCACCTTGTGTTGT			
**ELF1α**	ELF1α FwOm	CAAGGATATCCGTCGTGGCA	327	AF498320	103.2
	ELF1α ReOm	ACAGCGAAACGACCAAGAGG			

Supplementary Table S1. Primer sequences, size, primer efficiency and accession numbers for TNFα (pro inflammatory), TGFβ (anti inflammatory) and ELF1α (reference gene).
